# Supplementary material for: TNIK signaling imprints CD8+ T cell memory formation early after priming
Source: Nat Commun. 2020 Apr 2;11:1632. doi: 10.1038/s41467-020-15413-7 (PMC7118140; doi:10.1038/s41467-020-15413-7)
Supplement: Supplementary file 1 — Supplementary Information [file 41467_2020_15413_MOESM1_ESM.pdf]

SUPPLEMENTARY INFORMATION

TNIK signaling imprints CD8$^+$ T cell memory formation early after priming

Jaeger-Ruckstuhl et al.

Supplementary Figure 1 |

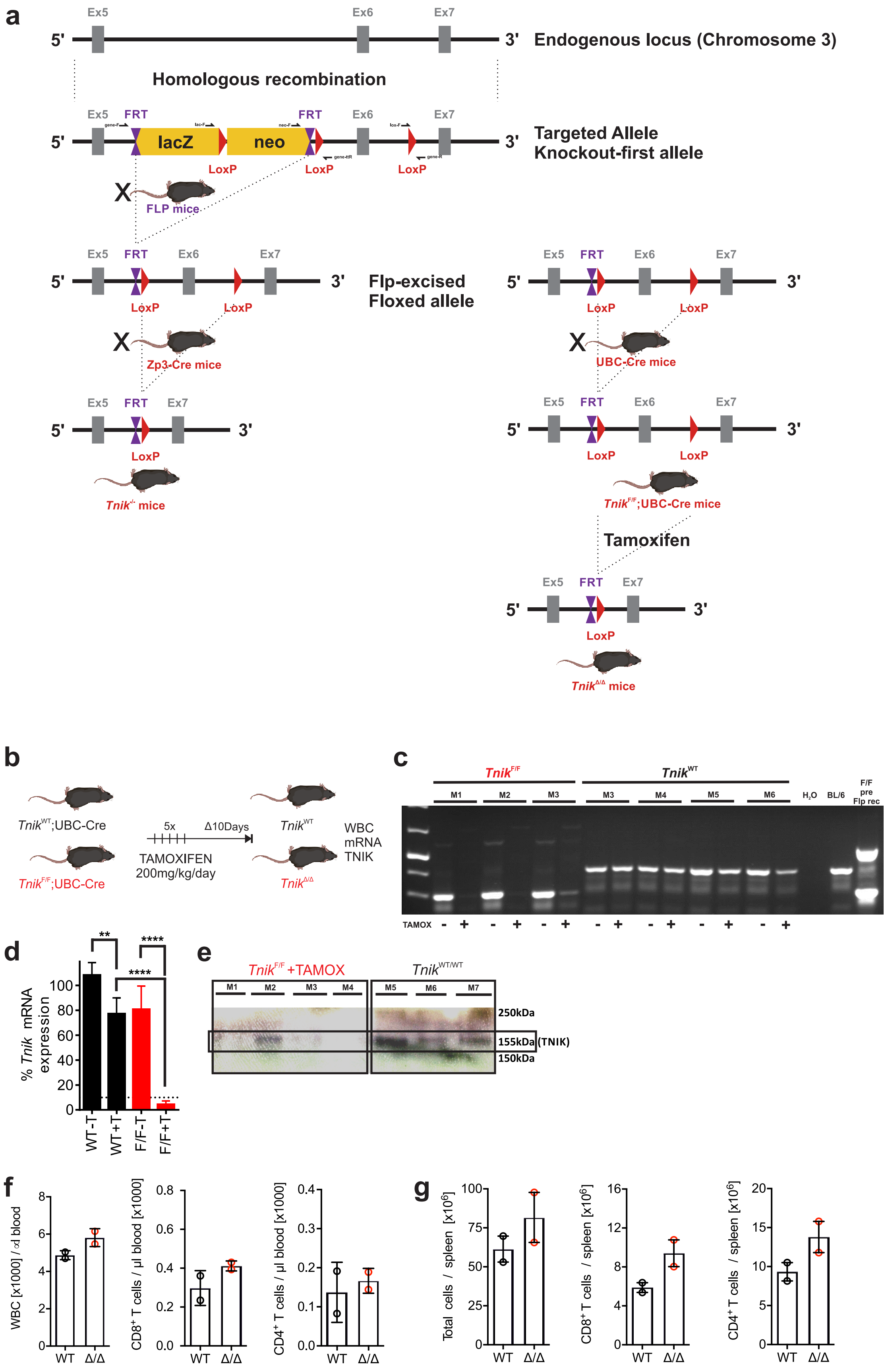

TNIK model generation and inducible *Tnik* deletion.

(a) The knockout-first allele was introduced into the endogenous locus in chromosome 3 by homologous recombination. Following FRT site recombination by FLP mice, the STOP cassette was excised, and TNIK was re-induced. Consequently $Tnik^{F/F}$ mice or littermate controls were crossed to Cre expressing mice (Zp3-Cre; constitutive deletion) or (tamoxifen inducible UBC-Cre; conditional deletion). **(b)** Experimental model of tamoxifen-induced *Tnik* deletion in $Tnik^{F/F}$;*UBC-Cre* mice ($Tnik^{\Delta/\Delta}$). Littermate controls $Tnik^{WT/WT}$;*UBC-Cre* mice were treated in parallel. **(c)** Genotyping, **(d)** relative quantification of gene expression > 10 days after treatment with Tamoxifen (T) and **(e)** western blot analysis of TNIK in blood lysates from $Tnik^{WT/WT}$ and $Tnik^{F/F}$ mice before (-T) and after (+T) Tamoxifen treatment. **(f-g)** Number of WBC, total splenocytes and $CD8^+$ and $CD4^+$ T cells in blood and spleen of $Tnik^{WT/WT}$ and $Tnik^{\Delta/\Delta}$ mice 18 days after last treatment dose of Tamoxifen. **(c,e)** Source data are provided as a Source Data file. Data are displayed as means ± SEM. Statistics: two-tailed students t-test, non-significant $P>0.05$, $^{**}P<0.01$, $^{****}P<0.0001$. Related to Fig. 1. Source data are provided as a Source Data file.

Supplementary Figure 2 |

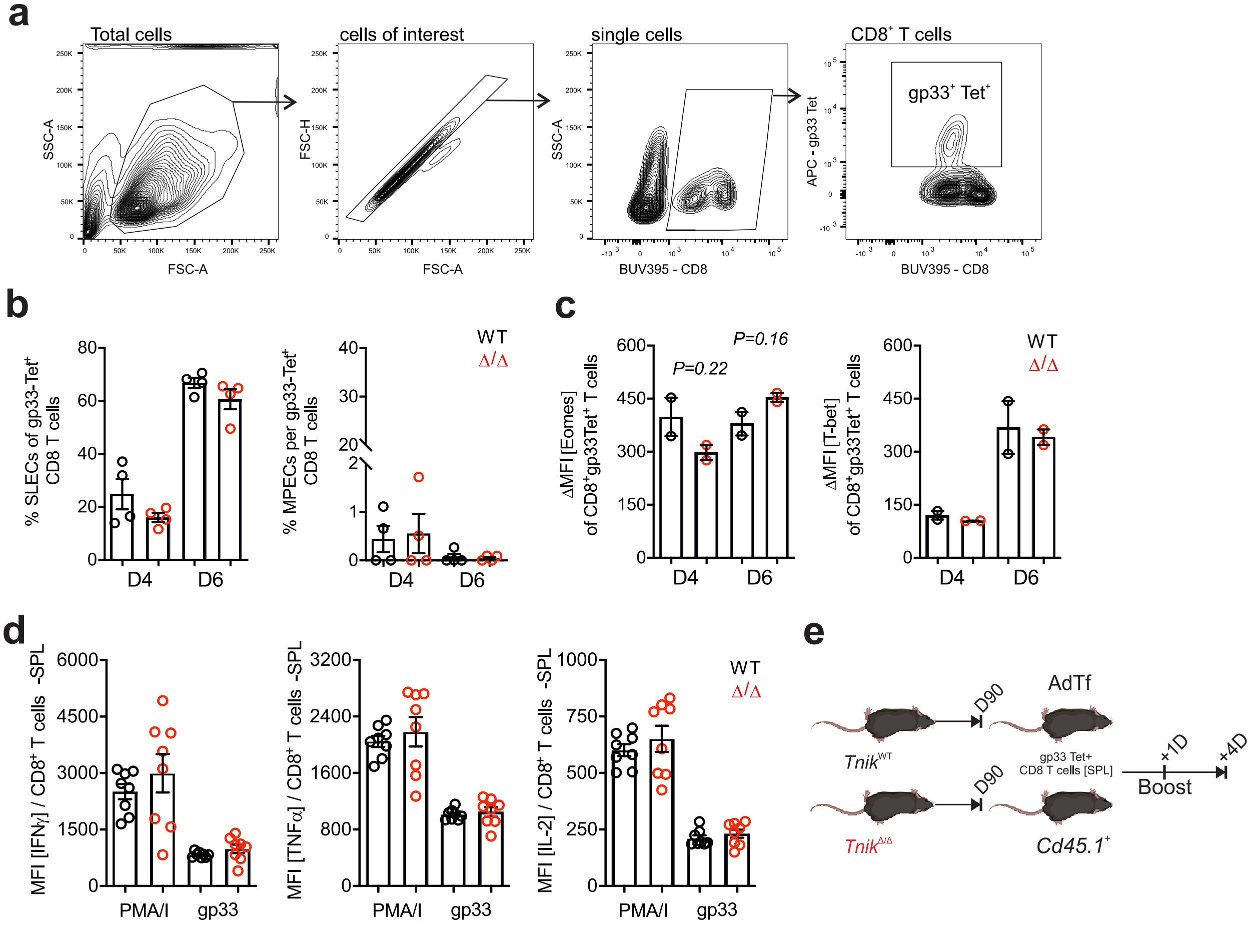

CD8⁺ T cell characterisation in mice with Tnik deletion pre LCMV priming.

(a) Gating strategy for gp33-Tet⁺ CD8⁺ T cells and (**b**) frequencies of SLECs (KLRG1⁺CD127⁻) and MPECs (KLRG1⁻CD127⁺) per gp33-Tet⁺ CD8⁺ T cells in the spleen of *Tnik*^{WT} and *Tnik*$^{\Delta/\Delta}$ mice 4 and 6 days after infection with 200 pfu LCMV. **(c)** Eomes and T-bet expression of gp33-Tet⁺ CD8⁺ T cells assessed 4 and 6 days p.i. (MFI normalized to isotype control (ΔMFI) of n=2 mice per time point). **(d)** CD8⁺ T cells from spleen of *Tnik*^{WT} and *Tnik*$^{\Delta/\Delta}$ mice 90 days p.i. were re-simulated with PMA/Ionomycin or gp33 peptide *in vitro* and delta MFI of TNFα, IFNγ and IL-2 are shown. **(e)** AdTf scheme of gp33-Tet⁺ memory CD8⁺ T cells into new congenic (*Cd45.1*⁺) recipients. Depicted: *Tnik*^{WT} (black bars), *Tnik*$^{\Delta/\Delta}$ (red bars). Data are displayed as means ± SEM. Statistics: two-tailed students t-test, non-significant *P>0.05*. Related to Fig. 1 and 2**.** Source data are provided as a Source Data file.

Supplementary Figure 3 |

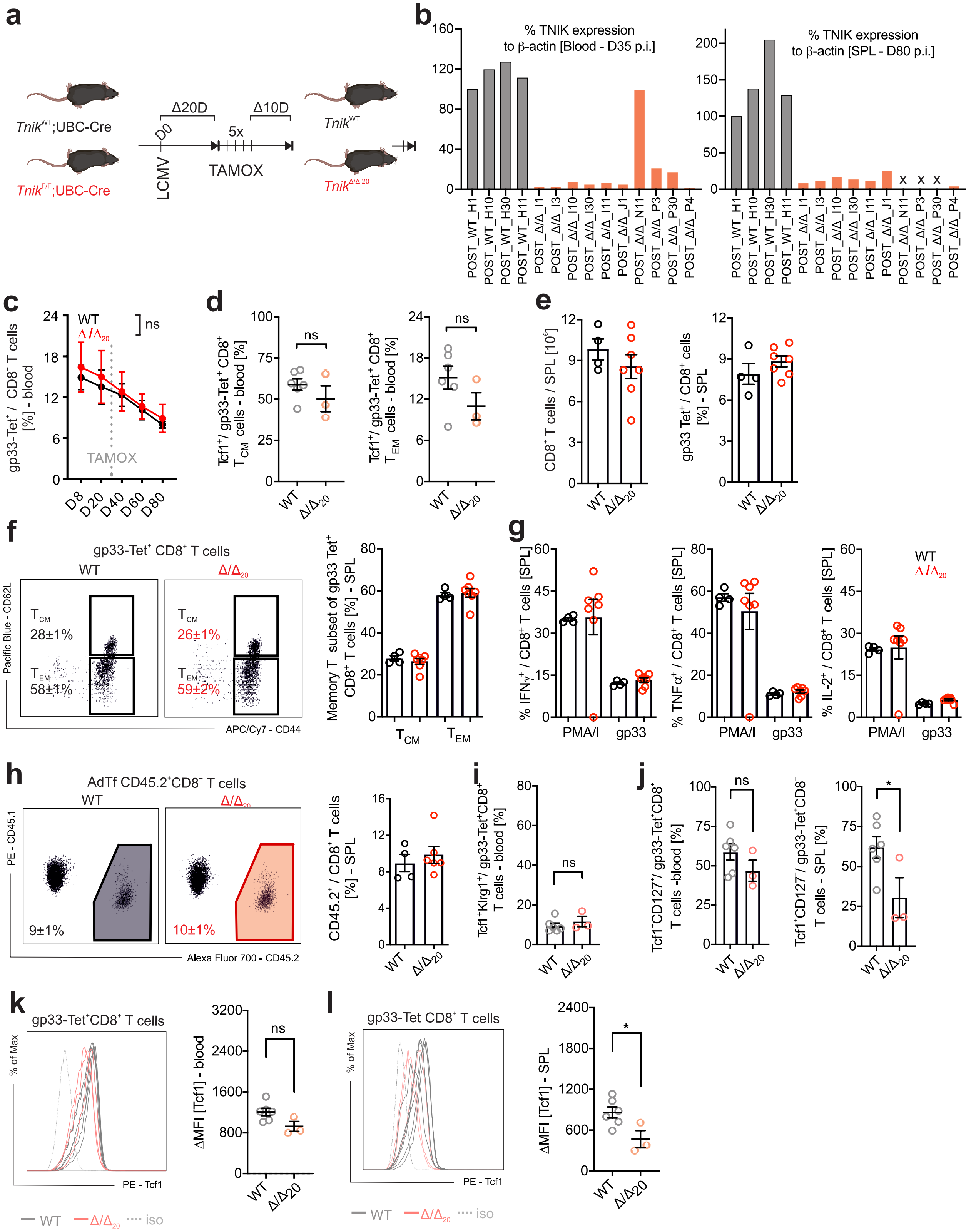

CD8$^+$ T cell characterisation in mice with Tnik deletion during contraction post LCMV infection.

(a) *Tnik*$^{F/F}$;*UBC-Cre* and *Tnik*$^{WT/WT}$;*UBC-Cre* mice were infected i.v. with 200pfu LCMV-WE and systemic deletion of TNIK was tamoxifen-induced during contraction phase between day 10 and 20 p.i. (*Tnik*$^{\Delta/\Delta\ 20}$). **(b)** Relative quantification of Tnik gene expression in PBMCs 10 days after last Tamoxifen treatment (left panel). Mice N11, P3 and P30 were excluded from the experiment due to insufficient deletion. Quantification of Tnik gene expression in FACS-purified gp33-Tet$^+$ memory CD8$^+$ T cells isolated from spleens 80 days p.i. (right panel). **(c)** Frequency of gp33-Tet$^+$ cells per CD8$^+$ T cells in blood in *Tnik*$^{WT/WT}$ and *Tnik*$^{\Delta/\Delta 20}$ mice over time and **(d)** Tcf1 expression of gp33-Tet$^+$ T_{CM} (CD44$^+$CD62L$^+$) and T_{EM} (CD44$^+$CD62L$^-$) cells in the blood on day 30 p.i. **(e)** CD8$^+$ T cell numbers in spleen and frequency of gp33-Tet$^+$ cells per CD8$^+$ T cells in spleen of mice sacrificed day 150 p.i.. **(f)** Dotplots and bar graphs showing distribution of gp33-Tet$^+$CD8$^+$ T_{CM} and T_{EM} cell subsets in spleen. **(g)** Frequencies of INFγ, TNFα or IL2-producing CD8$^+$ T cells after re-stimulation with PMA/Ionomycin or gp33-peptide. **(h)** AdTf of 3x10^4 FACS-purified gp33-Tet$^+$ CD8$^+$ T cells from spleens of day 150 memory mice (*Cd45.2*) injected into congenic recipient mice (*Cd45.1*$^+$) prior to re-infection with 200 pfu LCMV-WE. Dotplots and bar graphs show relative fraction of endogenous vs AdTf CD8$^+$ T cells 4 days p.i.. Frequencies of Tcf1 expressing Klrg$^+$ and CD127$^+$ gp33-Tet$^+$CD8$^+$ T cell subsets in blood or spleen **(i)** 4 days or **(j)** 38 days post rVV-G2 infection. **(k,l)** Histogram and ΔMFI of Tcf1 expressing gp33-Tet$^+$ CD8$^+$ T cells in blood and spleen day 38 post re-challenge. Depicted: *Tnik*^{WT} (gray lines/circles), *Tnik*$^{\Delta/\Delta 20}$ (red lines/circles), isotype controls (gray lines). Data is representative for one experiment of **(c, e-h)** n=4-7 mice or **(d,i-l)** n=3-6 mice. Data are displayed as means ± SEM. Statistics: **(c)** two-way ANOVA, **(d-l)** two-tailed students *t*-test, non-significant $P>0.05$, $^*P<0.05$. Related to Fig. 2. Source data are provided as a Source Data file.

Supplementary Figure 4 |

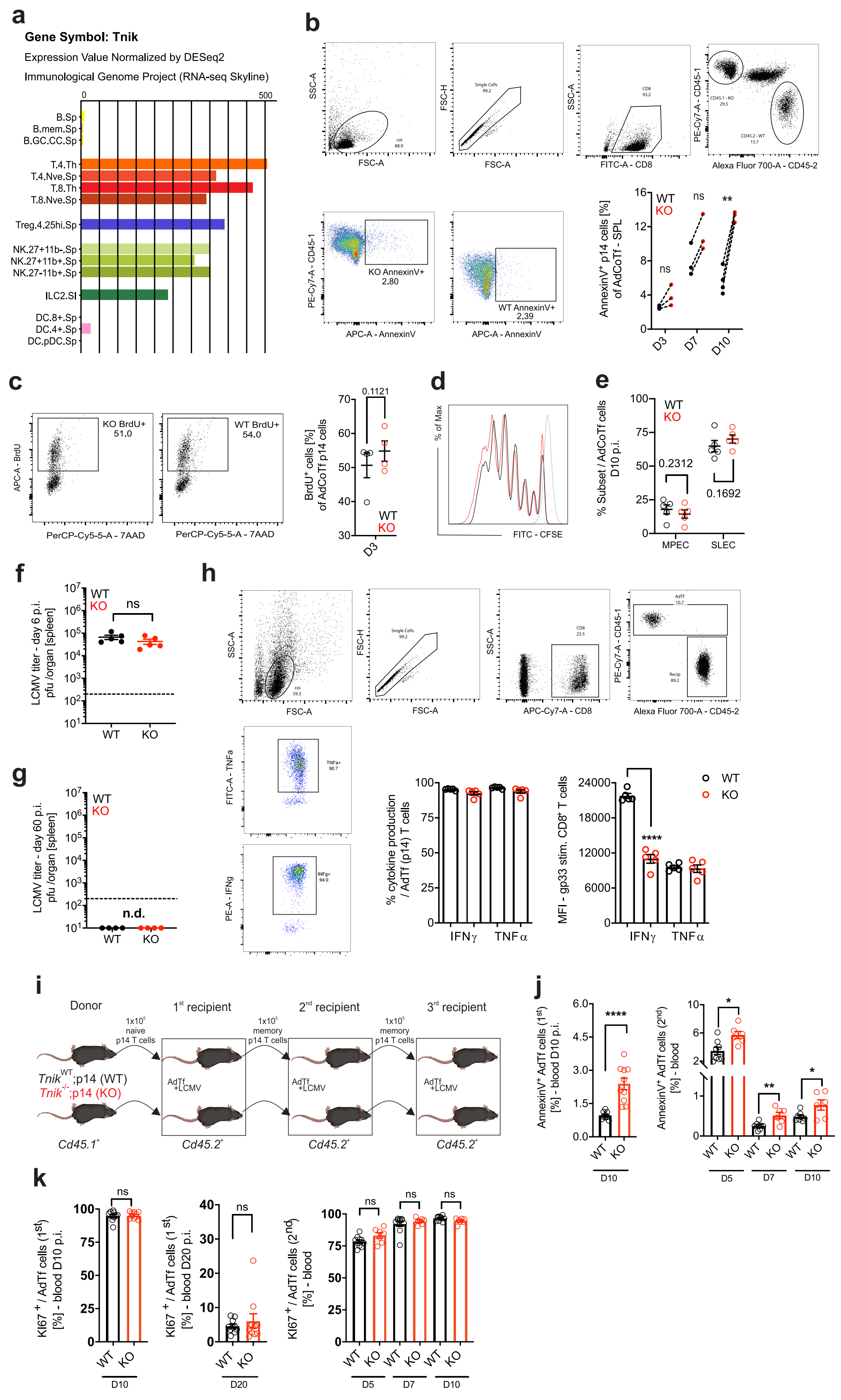

***Tnik* gene expression in immune cells and its role for $CD8^+$ T cell memory formation.** **(a)** TNIK gene expression analysis based on the negative binomial distribution (DESeq2) in subsets of immune cell subsets, such as B cells, $CD4^+$ / $CD8^+$ T cells, regulatory (T_{reg}) T cells, natural killer (NK) cells, innate lymphoid cells (ILCs) and dendritic cells (DCs) in spleen. **(b)** Gating strategy of AdCoTf p14 T cells and frequencies of $AnnexinV^+$ cells of AdCoTf p14 T cells at the indicated time points p.i.. **(c,d)** Gating after 3h BrdU incorporation and CFSE dilution of AdCoTf WT and KO p14 T cells 3 and 4 days after LCMV infection (10^4 pfu); non-activated ($CFSE^{high}$) control T cells (dotted line). **(e)** Frequency of MPECs and SELCs per total AdCoTf p14 T cells 10 days p.i.. **(f,g)** LCMV titer in spleen 6 and 60 p.i. with 10^4 pfu LCMV (n.d., non-detectable). **(h)** Gating strategy of AdTf p14 T cells and *in vitro* re-stimulation of AdTf p14 T cells from spleen of memory mice day 60 p.i.. Frequency of TNFα, IFNγ producing p14 T cells and MFI is shown. **(i)** Serial p14 T cell re-transfer model: $1x10^5$ donor p14 T cells (*$Cd45.1^+$*) were AdTf into primary (1st) recipients (*$Cd45.2^+$*) and infected with 10^4 pfu LCMV. Day 40 p.i., memory p 14 T cells form spleen were FACS-sorted, pooled and re-transferred ($1x10^5$/mouse) into secondary (2nd) and tertiary (3rd) recipients (*$Cd45.2^+$*), respectively, previous to LCMV infection. **(j,k)** Frequencies of $AnnexinV^+$ and $Ki67^+$ cells per AdTf p14 T cells at the indicated time points during primary and secondary immune response. Depicted: WT p14 (black lines/circles), KO p14 (red lines/circles). Data are displayed as means ± SEM. Statistics: two-tailed students t-test, non-significant P>0.05, *P<0.05, **P<0.01, ****P<0.0001. Related to Fig. 3. Source data are provided as a Source Data file.

Supplementary Figure 5 |

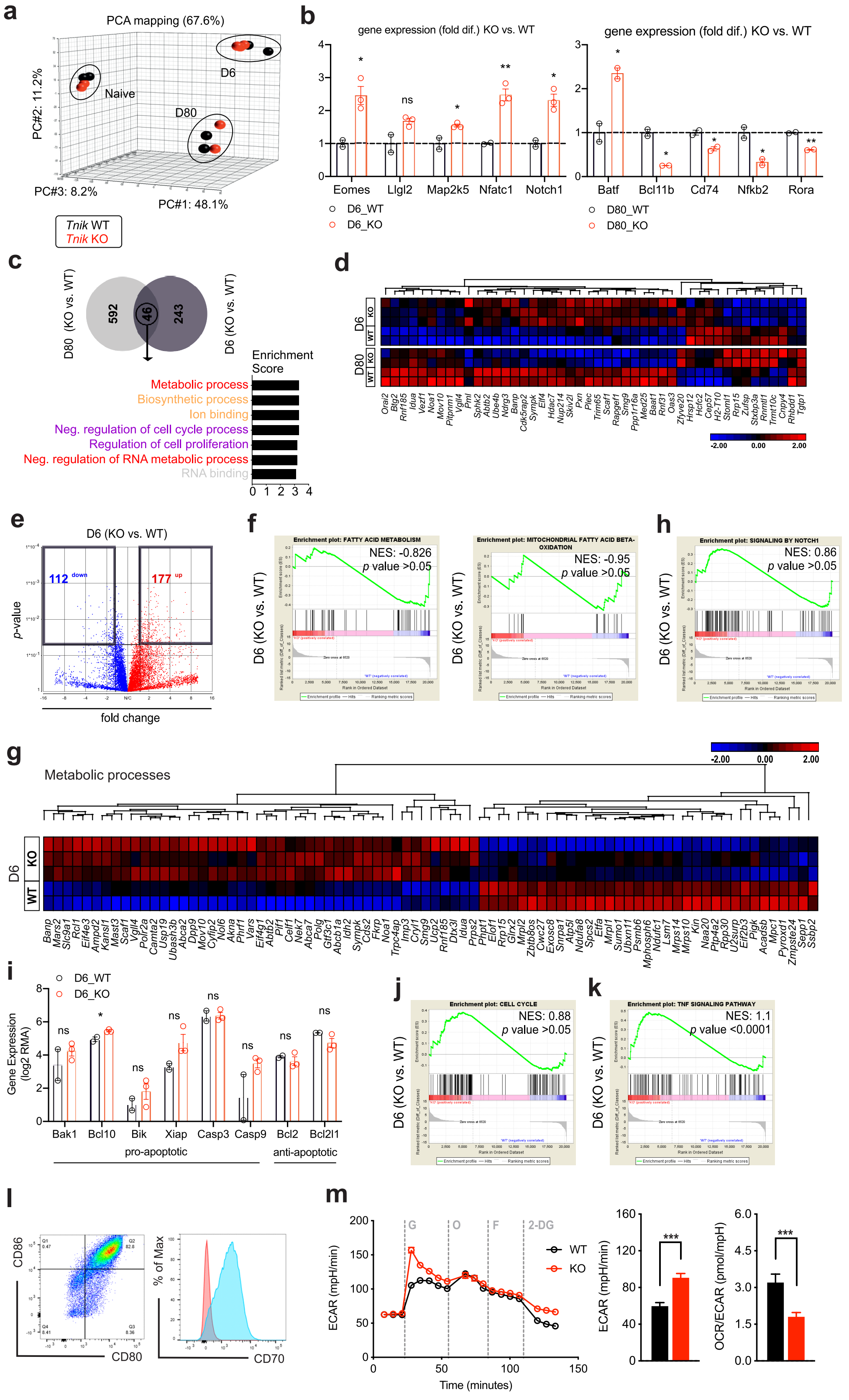

Gene expression analysis of WT and KO p14 T cells 6 days after LCMV infection.

(a) Principal component analysis (PCA) of normalized gene expression counts in WT (black) and KO (red) p14 T cells at the time points indicated. **(b)** Confirmation of selected differentially expressed genes in D6 and D80 WT and KO p14 T cells by RTqPCR **(c)** Venn-diagram of differentially expressed genes in D6 and D80 KO vs WT p14 T cells**,** GO analysis and **(d)** heat map of the 46 differentially expressed intersection genes. **(e)** Volcano plot comparing the normalized expression of gene probe sets of D6 KO vs WT p14 T cells; y-axis – negative log of p value; x-axis – log2-transformed fold change. **(f)** Gene set enrichment analysis (GSEA) of fatty acid metabolism and mitochondrial fatty acid beta-oxidation using profiles of the running Normalized Enrichment Scores (NES) and positions of gene set members on the rank ordered list in GSEA. **(g)** Heat map showing differentially expressed genes between WT and KO effector p14 T cells involved in metabolism. **(h,j-k)** Gene set enrichment analysis (GSEA) of signaling by Notch1, cell cycle and TNF signaling pathway using profiles of the running Normalized Enrichment Scores (NES) and positions of gene set members on the rank ordered list in GSEA. **(i)** Gene expression of selected pro- and anti-apoptotic genes of D6 WT and KO p14 effectors. **(l)** Dot plot showing maturation ($CD80^+CD86^+$) status and histogram showing CD70 expression of H8-DCs assessed by flow-cytometry. **(m)** Extracellular metabolic flux analysis showing extracellular acidification rate (ECAR) of day 3 activated (H8-DCs) WT and KO p14 T cells. Bar graphs: ECAR represents the basal glycolysis as determined using glycolysis stress test. OCR/ECAR ratios were calculated for basal glycolysis and respiration. G, glucose; O, oligomycin; F, FCCP; 2-DG, 2-deoxyglucose. Depicted: WT p14 (black lines/circles), KO p14 (red lines/circles). Data are displayed as means ± SEM. Statistics: two-tailed students t-test, non-significant $P>0.05$, $^*P<0.05$, $^{**}P<0.01$, $^{***}P<0.001$. Related to Fig. 4. Source data are provided as a Source Data file.

Supplementary Figure 6 |

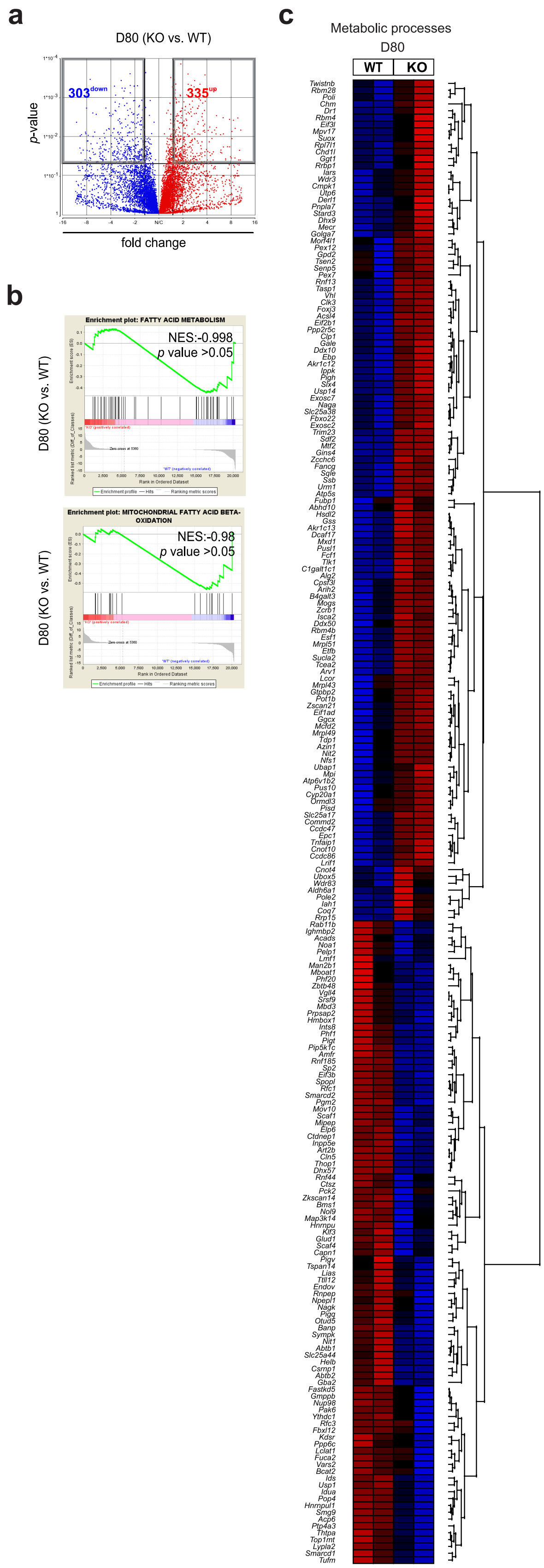

Gene expression analysis of WT and KO p14 T cells 80 days after LCMV infection. **(a)** Volcano plot comparing the normalized expression of gene probe sets of D80 p14 T cells (KO vs WT); y-axis – negative log of p value; x-axis – log2-transformed fold change. **(b)** Gene set enrichment analysis (GSEA) of fatty acid metabolism and mitochondrial fatty acid beta-oxidation using profiles of the running Normalized Enrichment Scores (NES) and positions of gene set members on the rank ordered list in GSEA. **(c)** Heat maps showing differentially expressed genes between WT and KO memory p14 T cells involved in metabolism. Related to Fig. 5.

Supplementary Figure 7 |

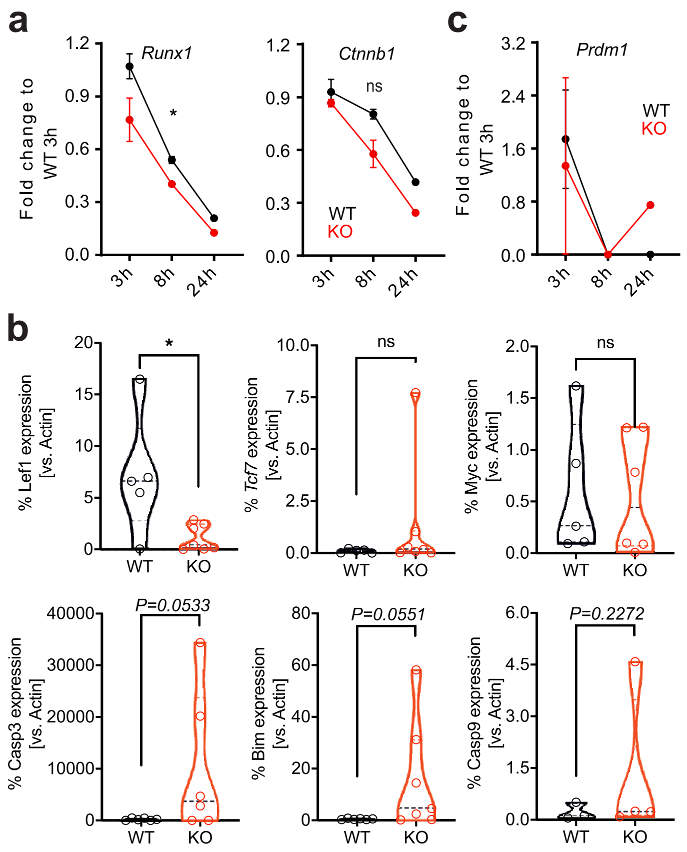

Gene expression of *in vitro* and *ex vivo* activated p14 T cells.

(a,c) Changes of *Runx1*, *Ctnnb1* and *Prdm1* gene expression in activated WT and KO p14 T cells at the time points indicated. **(b)** Normalized *Lef1*, *Tcf7*, *Myc*, *Casp3*, *Bim* and *Casp9* gene expression analysis of AdTf WT and KO p14 T cells isolated form the spleen 48h post LCMV infection. Each dot represents pooled cells of 2-3 mice (n=4-7). Depicted: WT p14 (black lines/circles), KO p14 (red lines/circles). Data are displayed as means ± SEM. Statistics: two-tailed students t-test, non-significant $P>0.05$, *$P<0.05$. Related to Fig. 6. Source data are provided as a Source Data file.

Supplementary Table 1 | Primers for quantitative Real-Time PCR, Related to Methods.

OLIGONUCLEOTIDES		
REAGENT or RESOURCE	SOURCE	IDENTIFIER
Actinb-F: AGATGACCCAGATCATGTTTGAG	This paper	N/A
Actinb-R: GTACGACCAGAGGCATACAG	This paper	N/A
Gapdh-F: AGAACATCATCCCTGCATCC	This paper	N/A
Gapdh-R: TCATCATACTTGGCAGGTTTCTC	This paper	N/A
Tnik-lox-F: CCAACTCTGGTTGGTTATGG	This paper	N/A
Tnik-lox-R: TGGTGCAGGTGACTCAG	This paper	N/A
Tcf7-F: CGCTGCATAACAAGCC	This paper	N/A
Tcf7-R: CCAGCTCACAGTATGGG	This paper	N/A
Lef1-F: ACCCTCCTACTCCAGTTACTC	This paper	N/A
Lef1-R: GGGCACTTTATTTGATGTCCTC	This paper	N/A
Myc-F: CAGTGGTCTTTCCCTACCC	This paper	N/A
Myc-R: TCTTGCTCTTCTTCAGAGTCG	This paper	N/A
Runx1-F: GAGGTACTAGCTGACCACCC	This paper	N/A
Runx1-R: TGCCACCACCTTGAAAGC	This paper	N/A
Msi2-F: CGCTATGGAGGCAAATGGG	This paper	N/A
Msi2-R: TAAGGCTATCTGGTGAGGTCTG	This paper	N/A
Ifng-F: CAAGTTTGAGGTCAACAACCC	This paper	N/A
Ifng-R: GAATCAGCAGCGACTCCT	This paper	N/A
Prdm1-F: GACGGGGGTACTTCTGTTCA	This paper	N/A
Prdm1-R: GGCATTCTTGGGAACTGTGT	This paper	N/A
Tbx21-F: CTCTCCACAAGTACCAGCC	This paper	N/A
Tbx21-R: GAGTGATCTCTGCGTTCTGG	This paper	N/A
Notch1-F: CAGACCAACACGCAGTACC	This paper	N/A
Notch1-R: CGTCAATGCCTCGCTTCTG	This paper	N/A
Ctnnb1-F: GCAAGTAGCTGATATTGACGG	This paper	N/A
Ctnnb1-R: CAAACTGCGTGGATGGGA	This paper	N/A
Eomes-F: TCTCTGCACAAATACCAACC	This paper	N/A
Eomes-R: CTTTAGCTGGGTGATATCCGT	This paper	N/A
Llgl2-F: AAAGCCAAGAAGCACAACC	This paper	N/A
Llgl2-R: CTTTAGGACCCAGGCGT	This paper	N/A
Map2k5-F: GTGGTCTCAGATTCGCTTCC	This paper	N/A
Map2k5-R: ATGATGTGCTTTGTAGACTGTG	This paper	N/A
Nfatc1-F: TATATGAGCCCATCCTTGCC	This paper	N/A
Nfatc1-R: GTCTCATAGTGAGCCCTGTG	This paper	N/A
Batf-F: CTAGTGAGAAGATCGCCCAG	This paper	N/A
Batf-R: CAGATGAGTCCTGTTTGCCA	This paper	N/A
Bcl11b-F: GTTCTCCCACCCTGTTTCC	This paper	N/A
Bcl11b-R: TCTTTACCTGACAACTCACACTG	This paper	N/A
Cd74-F: CATCTGCTCACGAGGTCTG	This paper	N/A
Cd74-R: GATCTTCCAGTTCACGCCA	This paper	N/A
Nfkb2-F: CATCCATGACAGCAAGTCTCC	This paper	N/A
Nfkb2-R: ATCATCCTCATAGAACCGAACC	This paper	N/A
Rora-F: CGAGATGCTGTCAAGTTTGG	This paper	N/A
Rora-R: ATTGGCTGAGATGTTGTAGGTG	This paper	N/A
Casp3-F: GAGCACTGGAATGTCATCTC	This paper	N/A
Casp3-R: ACAGGCCCATTTGTCCC	This paper	N/A
Casp9-F: GCAGATATGGCATACACCC	This paper	N/A
Casp9-R: GTGCTCAAGTTTGTCACGG	This paper	N/A
Bim-F: ACAGAACCGCAAGCTTCC	This paper	N/A
Bim-R: CCCTCCTTGTGTAAGTTTCGT	This paper	N/A
