# Supplementary material for: TNIK signaling imprints CD8+ T cell memory formation early after priming
Source: Nat Commun. 2020 Apr 2;11:1632. doi: 10.1038/s41467-020-15413-7 (PMC7118140; doi:10.1038/s41467-020-15413-7)
Supplement: Supplementary file 3 — Description of Additional Supplementary Files [file 41467_2020_15413_MOESM3_ESM.pdf]

Description of Additional Supplementary Files

Supplementary Data 1 | Differentially expressed genes between WT and KO p14 $CD8^+$ T cells 6 days p.i. (*p* adj <0.05 and fold differences ≥ 1.5), Related to Fig. 4

Supplementary Data 2 | Differentially expressed genes between WT and KO p14 $CD8^+$ T cells 80 days p.i. (*p* adj <0.05 and fold differences ≥ 1.5), Related to Fig. 5

Supplementary Data 3 | Images of representative dividing cells after *in vitro* activation, Related to Fig. 7
