# Supplementary material for: TNIK signaling imprints CD8+ T cell memory formation early after priming
Source: Nat Commun. 2020 Apr 2;11:1632. doi: 10.1038/s41467-020-15413-7 (PMC7118140; doi:10.1038/s41467-020-15413-7)
Supplement: Supplementary file 7 — Reporting Summary [file 41467_2020_15413_MOESM7_ESM.pdf]

nature research

Corresponding author(s): Adrian F. Ochsenbein

Last updated by author(s): Jan 6, 2020

Reporting Summary

Nature Research wishes to improve the reproducibility of the work that we publish. This form provides structure for consistency and transparency in reporting. For further information on Nature Research policies, see Authors & Referees and the Editorial Policy Checklist.

Statistics

For all statistical analyses, confirm that the following items are present in the figure legend, table legend, main text, or Methods section.

n/a	Confirmed	
☐	☒	The exact sample size (*n*) for each experimental group/condition, given as a discrete number and unit of measurement
☐	☒	A statement on whether measurements were taken from distinct samples or whether the same sample was measured repeatedly
☐	☒	The statistical test(s) used AND whether they are one- or two-sided<br>*Only common tests should be described solely by name; describe more complex techniques in the Methods section.*
☒	☐	A description of all covariates tested
☒	☐	A description of any assumptions or corrections, such as tests of normality and adjustment for multiple comparisons
☐	☒	A full description of the statistical parameters including central tendency (e.g. means) or other basic estimates (e.g. regression coefficient) AND variation (e.g. standard deviation) or associated estimates of uncertainty (e.g. confidence intervals)
☐	☒	For null hypothesis testing, the test statistic (e.g. *F*, *t*, *r*) with confidence intervals, effect sizes, degrees of freedom and *P* value noted<br>*Give P values as exact values whenever suitable.*
☒	☐	For Bayesian analysis, information on the choice of priors and Markov chain Monte Carlo settings
☒	☐	For hierarchical and complex designs, identification of the appropriate level for tests and full reporting of outcomes
☒	☐	Estimates of effect sizes (e.g. Cohen's *d*, Pearson's *r*), indicating how they were calculated

Our web collection on statistics for biologists contains articles on many of the points above.

Software and code

Policy information about availability of computer code

Data collection	Gene Skyline platform (http://www.immgen.org)
Data analysis	Flow cytometric data was analyzed FlowJo (V.10.0.8, TreeStar, Inc.) software<br>ImageStream X® analysis was performed using Amnis IDEAS® software<br>RNA-seq data Array Star Software v13<br>Ariadne Genomic Pathway Studio® software<br>Gene set enrichment analysis software v3.0

For manuscripts utilizing custom algorithms or software that are central to the research but not yet described in published literature, software must be made available to editors/reviewers. We strongly encourage code deposition in a community repository (e.g. GitHub). See the Nature Research guidelines for submitting code & software for further information.

Data

Policy information about availability of data

All manuscripts must include a data availability statement. This statement should provide the following information, where applicable:

- Accession codes, unique identifiers, or web links for publicly available datasets
- A list of figures that have associated raw data
- A description of any restrictions on data availability

The NGS data are available in the NCBI GEO database under the accession code: GSE127734
Related: Figures 4 & 5, Supplementary Figures 5 & 6

Field-specific reporting

Please select the one below that is the best fit for your research. If you are not sure, read the appropriate sections before making your selection.

☒ Life sciences ☐ Behavioural & social sciences ☐ Ecological, evolutionary & environmental sciences

For a reference copy of the document with all sections, see nature.com/documents/nr-reporting-summary-flat.pdf

Life sciences study design

All studies must disclose on these points even when the disclosure is negative.

Sample size	No sample size calculations have been performed. Sample size was determined according to standard practice in the field.
Data exclusions	Only two data points were excluded due to technical acquisition error (Flow cytometry) > Fig 2h,j (WT, spleen)
Replication	Two or more independent experiments were performed or sufficient sample sizes were involved.
Randomization	Mice analyzed were litter mates, age and sex-matched whenever possible.
Blinding	Investigators were not blinded to mouse genotypes during experiments.<br>Data reported for mouse experiments are based on quantitative flow cytometry.

Reporting for specific materials, systems and methods

We require information from authors about some types of materials, experimental systems and methods used in many studies. Here, indicate whether each material, system or method listed is relevant to your study. If you are not sure if a list item applies to your research, read the appropriate section before selecting a response.

Materials & experimental systems

n/a	Involved in the study	
☐	☒	Antibodies
☐	☒	Eukaryotic cell lines
☒	☐	Palaeontology
☐	☒	Animals and other organisms
☒	☐	Human research participants
☒	☐	Clinical data

Methods

n/a	Involved in the study	
☒	☐	ChIP-seq
☐	☒	Flow cytometry
☒	☐	MRI-based neuroimaging

Antibodies

Antibodies used

Anti-Mouse:
Anti-CD8 (53-6.7) PE eBioscience, Waltham, MA, USA Cat#:12-0081-83_1:200
Anti-CD8 (53-6.7) FITC BioLegend, San Diego, CA, USA Cat#:100706_1:600
Anti-CD8 (53-6.7) APC-Cy7 BioLegend, San Diego, CA, USA Cat#:100714_1:300
Anti-CD8 (53-6.7) APC BioLegend, San Diego, CA, USA Cat#:100711_1:50
Anti-CD8 (53-6.7) Alexa Fluor 700 BioLegend, San Diego, CA, USA Cat#:100730_1:800
Anti-CD8 (53-6.7) BUV395 BD Biosciences, Cat#: 563786; 1:600
Anti-CD4 (GK1.5) PE-Cy7 BioLegend, San Diego, CA, USA Cat#:100422_1:600
Anti-CD19 (6D5) APC-Cy7 BioLegend, San Diego, CA, USA Cat#:115530_1:300
Anti-CD11c (N418) FITC BioLegend, San Diego, CA, USA Cat#:117305_1:300
Anti-CD86 (GL1) APC BioLegend, San Diego, CA, USA Cat#:105011_1:200
Anti-CD80 (16-10A1) Pacific Blue BioLegend, San Diego, CA, USA Cat#:104723_1:200
Anti-CD45.2 (104) Alexa Fluor 700 BioLegend, San Diego, CA, USA Cat#:109822_1:100
Anti-CD45.1 (A20) PE-Cy7 BioLegend, San Diego, CA, USA Cat#:110730_1:200
Anti-CD45.1 (A20) PerCP-Cy5.5 BioLegend, San Diego, CA, USA Cat#:110728_1:200
Anti-CD45.1 (A20) PE eBioscience, Waltham, MA, USA Cat#:12-0453-82_1:200
Anti-CD62L (MEL-14) Pacific Blue BioLegend, San Diego, CA, USA Cat#:104423_1:200
Anti-CD62L (MEL-14) Alexa Fluor 700 BioLegend, San Diego, CA, USA Cat#:104426_1:200
Anti-CD44 (IM7) APC-Cy7 BioLegend, San Diego, CA, USA Cat#:103028_1:100
Anti-KLRG1 (2F1/KLRG1) PE-Cy7 BioLegend, San Diego, CA, USA Cat#:138415_1:300
Anti-KLRG1 (2F1/KLRG1) Biotin BioLegend, San Diego, CA, USA Cat#:138405_1:100
Brilliant Violet 510 Streptavidin BioLegend, San Diego, CA, USA Cat#:405234_1:500
Anti-CD127 (A7R34) FITC eBioscience, Waltham, MA, USA Cat#:11-1271_1:100
Anti-CD70 (FR70) PE eBioscience, Waltham, MA, USA Cat#:12-0701-83_1:300
Anti-TCR Va2 (B20.1) PE BioLegend, San Diego, CA, USA Cat#:127807_1:200

Anti-TNFa (MP6-XT22) FITC eBioscience, Waltham, MA, USA Cat#:11-7321-82_1:300
Anti-IFNg (XMG1.2) PE eBioscience, Waltham, MA, USA Cat#:12-7311-82_1:300
Anti-IL-2 (JES6-5H4) APC eBioscience, Waltham, MA, USA Cat#:17-7021-81_1:300
Anti-Eomes (Dan11mag) PE-Cy7 eBioscience, Waltham, MA, USA Cat#:25-4875-80_1:200
Anti-T-bet (4B10) PerCP-Cy5.5 BioLegend, San Diego, CA, USA Cat#:644805_1:200
Anti-GranzymeB (2E7) Pacific Blue BioLegend, San Diego, CA, USA Cat#:515407_1:200
Anti-Tcf7/Tcf1 (S33-966) PE BD Biosciences, Cat#:564217_1:100
AnnexinV PE ImmunoTools, Germany Cat#:31490014X2_1:100

Anti-Human:
Anti-CD27 (MT-271) APC-Cy7 BioLegend, Cat#:356423_1:100
Anti-CD8 (HIT8a) APC BioLegend, Cat#:300911_1:200

Functional assays:
Anti-mouse CD70 InVivoMAb (FR70) BioXCell, West Lebanon, NH, USA Cat#:BE0022_300ug/mouse (in vivo)
Anti-mouse CD70 InVivoMAb (FR70) BioXCell, West Lebanon, NH, USA Cat#:BE0022_20ug/ml (in vitro)
Anti-human CD28 (TGN1412) Generated in house_1ug/ml (coated)
Anti-CD27 (Varlimumab, 1F5) Generated in house_2ug/ml (coated)
Anti-CD3 (OKT3) Ultra LEAF Purified, BioLegend, Cat#:317347_1ug/ml (coated)
Human blocking anti-CD27 (1A4CD27) Beckman Coulter, Indianapolis, USA Cat#:PN IM2034_10ug/ml (soluble)
Anti-CD3 (OKT3) BioXCell, West Lebanon, NH, USA Cat#:BE0001-22_0.3ug/ml (coated)

Image Stream/Immunofluorescence:
Rabbit-anti-TNIK (D-16) Santa Cruz Biotec., Dallas, TX, USA Cat#:sc-100205_1:50
Mouse-Anti-active-b-catenin (8E7) Merck Millipore, Burlington, MA, USA Cat#:05-665_1:100
Rabbit-anti-Numb Abcam, Cambridge, MA, USA Cat#:ab14140_1:25
Rabbit-anti-Numb Abcam, Cambridge, MA, USA Cat#:ab4147_1:20
Mouse-anti-a-tubulin Abcam, Cambridge, MA, USA Cat#:ab7291_1:200
Goat-anti-rabbit Alexa Fluor 568 Abcam, Cambridge, MA, USA Cat#:ab175695_1:400
Goat-anti-rabbit Alexa Fluor 647 Abcam, Cambridge, MA, USA Cat#:ab150083_1:100
Goat-anti-mouse Alexa Fluor 546 Invitrogen, Carlsbad, CA, USA Cat#:A-11030_1:100
Biotinylated goat-anti-rabbit Invitrogen, Carlsbad, CA, USA Cat#:65-6140_1:5000
Biotinylated goat-anti-mouse Invitrogen, Carlsbad, CA, USA Cat#:31800_1:500
Streptavidin-Cy2 Jackson ImmunoResearch, Cat#:016-220-084_1:200
Streptavidin-Cy5 Jackson ImmunoResearch, Cat#:016-170-084_1:200

Plaque Forming Assay:
VL4 Rat-Anti-LCMV Antibody, Generated in house_undiluted supernatant
Peroxidas-conjugated AffiniPure Goat Anti-Rat IgG Dianova, Hamburg, Germany Cat#:112-035-003_1:400

Western Blot:
TNIK Polyclonal Antibody Thermo Fisher Scientific, Waltham, MA, USA Cat#:PA1-20639_1:200
Monoclonal Anti-b-Actin Antibody (AC-15) Sigma Aldrich, St. Louis, MO, USA Cat#:A3854_1:50000
(HRP)-conjugated goat-anti-rabbit IgG Thermo Fisher Scientific, Waltham, MA, USA Cat#:31460_1:2000

Validation

Antiboodies were tested on spenocytes isolated from naive C57BL6/J mice or human PBMCs and titrated.

Eukaryotic cell lines

Policy information about cell lines

Cell line source(s)

TM-LCL obtained form Prof. Greenberg, Seattle, USA.
MC57 purchased form ATCC.

Authentication

TM-LCLs were stained positive for CD19, a marker expressed on the lymphoblastoid B cell line.
MC57, no authentication was performed.

Mycoplasma contamination

These cell lines were mycoplasma free.

Commonly misidentified lines
(See ICLAC register)

No misidentified lines were used.

Animals and other organisms

Policy information about studies involving animals; ARRIVE guidelines recommended for reporting animal research

Laboratory animals

Mouse strains were all on C57BL/6J background. All mice included in experiments were sex (f/m) and age matched.

Wild animals

none

Field-collected samples

none

Ethics oversight	Animal experiments were approved by the local experimental animal committee of the Canton of Bern and performed according to Swiss laws for animal protection

Note that full information on the approval of the study protocol must also be provided in the manuscript.

Flow Cytometry

Plots

Confirm that:

☒ The axis labels state the marker and fluorochrome used (e.g. CD4-FITC).

☒ The axis scales are clearly visible. Include numbers along axes only for bottom left plot of group (a 'group' is an analysis of identical markers).

☒ All plots are contour plots with outliers or pseudocolor plots.

☒ A numerical value for number of cells or percentage (with statistics) is provided.

Methodology

Sample preparation	Surface staining:<br>Ex vivo isolated cells were surface stained for a minimum of 30 min at 4°C in FACS buffer.<br>Intracellular staining:<br>Ex vivo isolated cells were surface stained for a minimum of 30 min at 4°C, washed in FACS buffer, permeabilized for 30 min at RT in BD Cytofix/CytopermTM solution (BD Bioscience), washed in BD Perm/Wash buffer (BD Bioscience) and intracellularly stained in BD Perm/Wash buffer (BD Bioscience) for 30 min at 4°C.
Instrument	Data acquisition was performed using a LSR Fortessa or a LSR II SORP (BD Biosciences) flow cytometer.<br>Cell-sorting was performed using FACS Aria or FACS Aria III (BD Bioscience) cell sorters.
Software	FlowJo (V.10.0.8, TreeStar, Inc.) software
Cell population abundance	A minimum of 400`000 cells / sample were aquired for flow cytometry.<br>5`000-500`000 cells were SORTed from splenocytes of immunized mice for further alaysis/re-transfer.
Gating strategy	FSC/SSC (cells of interest) > FSC-H/FSC-A (doublet exclusion) > SSC/CD8 (CD8 T cells) > gp33-Tet/CD8 (in conditional depletion experiments) or CD45.1/CD45.2 (in AdTf/AdCoTf experiments) > diverse other markers.

☒ Tick this box to confirm that a figure exemplifying the gating strategy is provided in the Supplementary Information.
